# Mechanism of induction of binucleated cells by multiwalled carbon nanotubes as revealed by live-cell imaging analysis

**DOI:** 10.1186/s41021-015-0003-y

**Published:** 2015-06-16

**Authors:** Manabu Yasui, Nagisa Kamoshita, Tetsuji Nishimura, Masamitsu Honma

**Affiliations:** Division of Genetics and Mutagenesis, National Institute of Health Sciences, 1-18-1 Kamiyoga, Setagaya-ku, Tokyo 158-8501 Japan; Faculty of Pharmaceutical Sciences, Teikyo Heisei University, 4-21-2 Nakano, Nakano-ku, Tokyo 164-8530 Japan

**Keywords:** Polyploidization, Crocidolite, Cytokinesis

## Abstract

**Introduction:**

Asbestos-induced formation of mesothelioma has been attributed to phenotypic and morphological changes in cells caused by polyploidization and aneuploidization, and multiwalled carbon nanotubes (MWCNTs) are suspected to have similar adverse effects due to the similarity in their physical form. MWCNTs and crocidolite, a kind of asbestos, show similar genotoxicity characteristics *in vitro*, including induction of binucleated cells. We here focused on the mechanisms underlying polyploidization during cell division on exposure to MWCNTs and conducted confocal live-cell imaging analysis using MDA-435 human breast cancer cells in which chromosomes and centromeres were visualized using fluorescent proteins.

**Findings:**

During anaphase, relatively short MWCNT fibers (approximately 5 μm) migrated rapidly to either of the daughter cells, whereas some long MWCNT fibers (approximately 20 μm) remained inside the contractile ring and induced the formation of binucleated cells through impairment of cytokinesis. This toxicity mechanism has also been observed with crocidolite.

**Conclusions:**

Our findings indicate that the mechanism of polyploidization by MWCNTs is very similar to that observed with crocidolite.

**Electronic supplementary material:**

The online version of this article (doi:10.1186/s41021-015-0003-y) contains supplementary material, which is available to authorized users.

## Introduction

Multiwalled carbon nanotubes (MWCNTs) have been suggested to be similar to crocidolite in terms of toxicity, given the similarity in their physical form [1–3]. Some animal studies have indicated that similar to crocidolite, intraperitoneally administered MWCNTs induce mesothelioma with a high frequency [4–6]. These results are generally consistent with the “Stanton–Pott hypothesis” that asbestos fibers with a diameter of ≤0.25 μm and length of ≥20 μm are highly carcinogenic [7, 8]. Muller *et al.* reported no increase in carcinogenesis in Wistar rats following intraperitoneal administration of short carbon nanotube (CNT) fibers whose length was less than that indicated in the hypothesis (average length: ≤1 μm) [9]. Only a limited number of *in vivo* studies have investigated the genotoxicity of MWCNTs [10, 11]. Kato *et al.* [11] performed intratracheal injection of MWCNTs (width: 70–110 nm, length: 1–4 μm) in wild-type ICR mice and found that the results of a comet assay, oxidative DNA adduct assay, and immunohistochemical analysis of nitric oxide synthase with the lung tissue were all positive. Therefore, the genotoxicity of MWCNTs has been shown to result predominantly from oxidative stress induced by excessive inflammatory responses to CNT fibers.

MWCNTs and asbestos show similar genotoxicity characteristics even in cell culture experiments, and both are known to induce polyploid cells (and multinucleated cells) with a high frequency [12–15]. Chromosomal abnormalities caused by polyploidization and aneuploidization alter the expression of a variety of genes involved in carcinogenesis and thus are believed to be closely related to asbestos-induced mesothelioma and bronchial cancer, as observed in animal studies [16, 17]. Jensen *et al.* conducted time-lapse analysis using a microscope applicable for live-cell observation to determine the mechanisms underlying the induction of abnormal binucleated and multinucleated cells by asbestos [18]. They observed that comparatively long crocidolite (15–50 μm) fibers were trapped in the contractile ring during anaphase in LLC-MK_2_ epithelial cells, which created a physical barrier to cytokinesis, eventually causing formation of binucleated cells. On the other hand, many reports have demonstrated a causal role of MWCNTs in cell multinucleation and polyploidization; however, only few have directly demonstrated the mechanism underlying the occurrence of these aberrations [19].

In this study, we conducted time-lapse analysis with a high-resolution confocal live-cell imaging system to elucidate the mechanism involved in the MWCNT-induced formation of binucleated cells using dichromatically visualized human cells in which chromosomes and centromeres were stained with different fluorescent proteins. We found that short CNT fibers (approximately 5 μm) migrated to either of the daughter cells immediately after chromosome segregation, whereas long fibers (approximately 20 μm) formed a bridge structure between the 2 daughter cells during anaphase and induced the formation of binucleated cells by impeding cytokinesis. This physical disruption of cytokinesis was very similar to the asbestos-induced disruption described above.

## Materials and methods

### MWCNTs

The MWCNTs used in this study were MWCNT-7 (Lot No.060125-01k) manufactured by Mitsui & Co., Ltd. (Ibaraki, Japan), which was same batch used in the study reported by Takagi *et al.* [4]. According to the report, these MWCNT fibers were approximately 100 nm in diameter and contained 27.5 % of MWCNTs ≥5 μm in length. The MWCNTs were suspended in 100 % fetal bovine serum (Gibco, Invitrogen, NY, USA) at a concentration of 1 mg/mL and were autoclaved for 15 min at 121 °C. Thereafter, Tween 80 (Tokyo Chemical Industry Co., Ltd., Tokyo, Japan) was added to a final concentration of 1.0 % in fetal bovine serum. The resulting mixture was subjected to ultrasonication using an ultrasonic homogenizer (VP30s, TAITEC Co., Saitama, Japan).

### Cell culture

Dichromatically visualized MDA-435 human breast cancer cells, in which chromosomes and centromeres were stained with a red fluorescent protein (mCherry–Histone H3 fusion) and green fluorescent protein (EGFP–CENP-A fusion), respectively, were kindly provided by Dr. Kenji Sugimoto (Osaka Prefecture University, Osaka, Japan) [20]. The cells were cultured at 37 °C (5 % CO_2_, 100 % humidity) in Dulbecco’s Modified Eagle’s medium (DMEM) (Nacalai Tesque, Kyoto, Japan), supplemented with 10 % fetal bovine serum. MDA-435 cell line, isolated from ductal adenocarcinoma of female breast, is aneuploid with most chromosome counts in the 55–60 range (modal number = 56) [21].

### Live-cell imaging

We used an FV1000 laser fluorescence microscope (Olympus Corp., Tokyo, Japan) equipped with a humid chamber to capture images as the cells were cultured. We also used a multi-Ar and He–Ne G laser and an objective lens with 60× magnification (1.20 Numerical Aperture). For imaging, 5 × 10^5^ MDA-435 cells were cultured in 2 mL of DMEM containing 10 % fetal bovine serum (37 °C, 5 % CO_2_, 100 % humidity) in a 35-mm glass base dish (IWAKI, ASAHI GLASS CO., Ltd., Tokyo, Japan). To minimize cytotoxicity of the laser, we conducted the experiments at a weak laser output such that ≥50 % cells divided after 24 h among the control cells. The acquired images were edited using Volocity Software (PerkinElmer Inc., Massachusetts, USA), and the resulting moving images were analyzed. When MWCNTs were added to the medium (final concentration: 0, 12.7, 25.3, or 50.6 μg/mL), images of a visual field containing a large number of cells in metaphase were taken at 5-min intervals for a period of 48–72 h (in the z-axial direction, photographs were taken every 2.0 μm). All cells in the visual field were counted for each MWCNT concentration, and the incidences (%) of cells that completed cell division, cells that were unable to undergo cell division and subsequently died, and cells that became binucleated were calculated by dividing the number of such cells by the total cell count. We did not take statistical analysis for the incidences of divided, dead, and bi-nucleated cells, since the images of a visual field containing a large number of cells in metaphase were intentionally selected. Bi- and multi-nucleated cells had more than two nuclei in a cell. The cell death was defined as mitotic catastrophe during M-phase (from prophase to telophase). Approximate length of MWCNT fiber was estimated from bar length given in each images.

## Results and discussion

### Endocytosis of MWCNTs

**Fig. 1:**
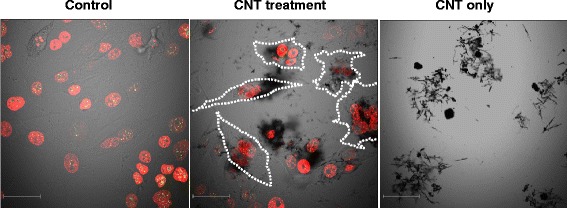
Comparison of untreated (control) and MWCNT (50.6 μg/mL) treated cells after 72 h. Many abnormal cells (such as multinucleated cells) were found in the MWCNT-treated cell group (the white dotted line in the image). Bar, 41 μm

We conducted time-lapse imaging at the MWCNT concentration of 50.6 μg/mL to determine how MWCNTs undergo endocytosis. The results showed that some cells actively ingested and incorporated CNT fibers within a few hours after addition of MWCNTs (Additional file 1, the right side in the movie) and other cells did not. Similar findings have been reported in a previous study in which MWCNTs were easily incorporated within 24 h in experiments with human neonatal epidermal keratinocytes [22]. CNT fibers that were incorporated into a cell remained there for a long time without being ejected and migrated to either of the daughter cells after cell division. In addition, we observed that CNT fibers were frequently stuck to the cell surface. After 72 h, virtually no abnormal cells (such as multinucleated cells) were found in the untreated (control) group of cells (Fig. 1). On the other hand, in the MWCNT-treated cell group, most cells were entangled with CNT fibers, and many multinucleated cells were observed (Fig. 1, the white dotted line).

### Cytotoxicity of MWCNTs and the incidence of binucleated cells

Time-lapse images of MDA-435 cells in the medium containing MWCNTs (0, 12.7, 25.3, or 50.6 μg/mL) were taken up to 72 h. The experiment was conducted 3 times with each concentration, and 232, 112, 170, and 282 cells were imaged in the visual field, respectively (Table 1). Among the untreated cells, 93 (40 %) of the 232 cells completed cell division and only 4 (1.7 %) cells died during cell division. In the MWCNT-treated group, 40 (38 %), 49 (27 %), and 34 (12 %) cells completed cell division and 4 (3.2 %), 11 (5.9 %), and 21 (7.6 %) cells died at concentrations of 12.7, 25.3, and 50.6 μg/mL, respectively. Thus, the number of cells that completed cell division decreased and the number of dead cells increased with an increase in MWCNT concentration, indicating the concentration-dependent cytotoxicity of MWCNTs. However, endocytosis of MWCNT was different depending on individual cells, as described above. Some cells did not undergo cell death when incorporated a small number of CNT fibers. Actually, both incidences of divided cells between control and low concentration (12.7 μg/mL) were almost same (40 and 38 %), as shown in Table 1. When ingested many CNT fibers even at low concentration, the cells gave rise to mitotic catastrophe. In addition, the number of binucleated cells was 0 (0 %), 5 (3 %), and 1 (0.4 %) at the respective concentrations. A possible reason for the lower incidence of binucleated cells at the highest dose of 50.6 μg/mL than at the lower dose of 25.3 μg/mL was that the cells underwent cell division less frequently at the highest dose, as described above; furthermore, a very large number of cells did not enter the mitotic phase and remained in interphase during imaging.

**Table 1:** Observation of cell division involving MWCNTs using live-cell imaging^a^

MWCNTs (μg/mL)	Experiment	Total of recorded cells	No. of divided cells	No. of dead cells during mitosis	No. of binucleated cells during mitosis
0	1	76	37	1	0
	2	74	27	2	0
	3	82	29	1	0
	Total	232	93 (40 %)	4 (1.7 %)	0
12.7	1	33	16	0	0
	2	32	13	1	0
	3	47	11	3	0
	Total	112	40 (38 %)	4 (3.2 %)	0
25.3	1	38	5	2	1
	2	51	19	2	2
	3	81	25	7	2
	Total	170	49 (27 %)	11 (5.9 %)	5 (3.0 %)
50.6	1	86	12	10	1
	2	101	11	5	0
	3	95	11	6	0
	Total	282	34 (12 %)	21 (7.6 %)	1 (0.4 %)

^a^Statistical analysis for the incidences of divided, dead, and binucleated cells was not done, as described in Materials and Methods

### Formation of binucleated cells through disturbance of cytokinesis

**Fig. 2:**
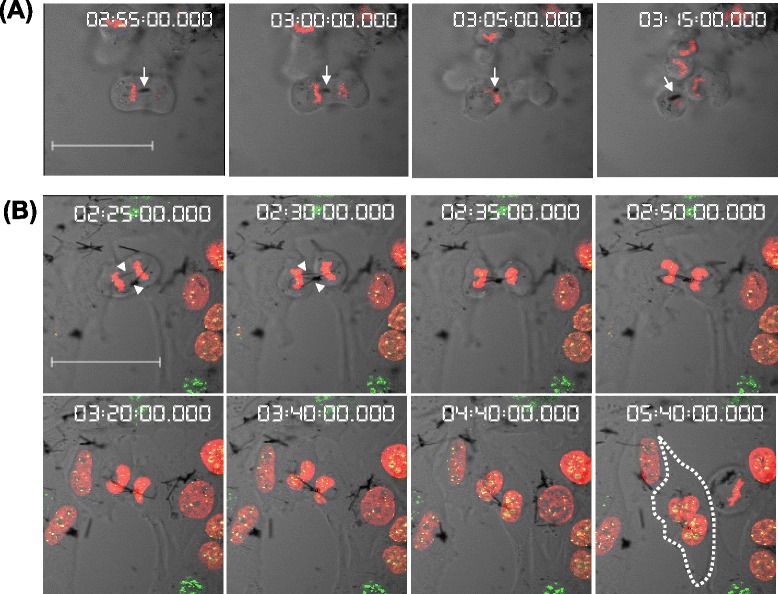
Time-lapse analysis of cell division involving MWCNT fibers (25.3 μg/mL). **a** Short MWCNT fibers (approximately 5 μm) did not inhibit cell division. **b** Long MWCNT fibers (approximately 20 μm) inhibited cytokinesis and induced the formation of binucleated cells (the white dotted line in the image at 5 h 40 min). Time (h:min) is shown at the top. Bar, 41 μm

Time-lapse images of typical cell division involving MWCNTs are shown in Fig. 2. Normal cell division was completed within 30 min of chromosome segregation during metaphase. Cell division involving short CNT fibers (approximately 5 μm) (Fig. 2a) occurred smoothly, similar to that in the untreated group of cells, and was completed within 30 min. These short CNT fibers migrated to the daughter cells immediately after chromosome segregation (the white arrow in Fig. 2a) and presumably exerted no lethal damage during karyokinesis or cytokinesis. In contrast, cell division involving long CNT fibers (approximately 20 μm) took almost 3 h (Fig. 2b). The long CNT fibers formed a bridge between the 2 daughter cells during anaphase (arrowheads in Fig. 2b), and remained in the contractile ring (2:30). Thereafter, karyokinesis was only slightly delayed and was completed normally without micronuclei formation (2:50). However, approximately at the same time, cytokinesis was impeded by the CNT fiber bridge; consequently, the constriction of the contractile ring was gradually abrogated (3:20). Thereafter, the borderline between the 2 cells disappeared (4:40), resulting in the formation of binucleated cells (5:40, the white dotted line). This disruption of cytokinesis was very similar to the process observed with crocidolite [18].

MWCNTs used in this experiment contained approximately 3500 ppm of iron and thus may have caused oxidative DNA damage to the cell genome by the reactive oxygen species formed during the Fenton reaction [4, 23, 24]. Nonetheless, even with the analysis system used in this study capable of detecting extremely small micronuclei [25], we did not observe any abnormality (such as micronuclei formation or abnormal multipolar division involving multiple centromeres) attributable to incubation with CNT fibers within the 72-h imaging period. Asakura *et al.* used MWCNT-7 (iron content: 4400 ppm) obtained from the same manufacturer to perform a chromosome abnormality test, an *in vitro* micronucleus test, and the *Hprt* gene mutation assay in Chinese hamster lung (CHL/IU) cells. The test results were all negative, but they observed an increase in the number of binucleated and polyploid cells with an increase in MWCNT concentration [14]. These results are consistent with the mechanism of induction of binucleated cells observed in this study and suggest that CNT fibers containing comparatively less iron result in greater physical disruption of cytokinesis than DNA damage by reactive oxygen species. In other words, MWCNTs may allow karyokinesis to proceed and may induce abnormal cells that are either binuclear or tetranuclear, considering that MWCNTs inhibit cytokinesis but do not cause lethal damage to the nucleus or chromosomes to the extent that prevents cell division (Fig. 1).

In conclusion, we observed that comparatively long MWCNTs (approximately ≥20 μm) inhibited cytokinesis during cell division and induced the formation of binucleated cells, whereas short MWCNTs did not. These results indicate that the mechanism of induction of binucleated cells by MWCNTs is very similar to that observed with crocidolite.

## Additional file

Additional file 1:
**Time-lapse of endocytosis of cells exposed to MWCNT (50.6 μg/mL).** MWCNTs were easily incorporated within a few hours. CNT fibers that were incorporated into a cell remained there for a long time without being ejected and migrated to either of the daughter cells after cell division. Bar, 41 μm.

## References

[1] Poland CA, Duffin R, Kinloch I, Maynard A, Wallace WA, Seaton A (2008). Carbon nanotubes introduced into the abdominal cavity of mice show asbestos-like pathogenicity in a pilot study. Nat Nanotechnol..

[2] Toyokuni S (2013). Genotoxicity and carcinogenicity risk of carbon nanotubes. Adv Drug Deliv Rev..

[3] Donaldson K, Poland CA, Murphy FA, MacFarlane M, Chernova T, Schinwald A (2013). Pulmonary toxicity of carbon nanotubes and asbestos - similarities and differences. Adv Drug Deliv Rev..

[4] Takagi A, Hirose A, Nishimura T, Fukumori N, Ogata A, Ohashi N (2008). Induction of mesothelioma in p53+/− mouse by intraperitoneal application of multi-wall carbon nanotube. J Toxicol Sci..

[5] Takagi A, Hirose A, Futakuchi M, Tsuda H, Kanno J (2012). Dose-dependent mesothelioma induction by intraperitoneal administration of multi-wall carbon nanotubes in p53 heterozygous mice. Cancer Sci..

[6] Sakamoto Y, Nakae D, Fukumori N, Tayama K, Maekawa A, Imai K (2009). Induction of mesothelioma by a single intrascrotal administration of multi-wall carbon nanotube in intact male Fischer 344 rats. J Toxicol Sci..

[7] Stanton MF, Layard M, Tegeris A, Miller E, May M, Morgan E (1981). Relation of particle dimension to carcinogenicity in amphibole asbestoses and other fibrous minerals. J Natl Cancer Inst..

[8] Pott F, Schlipkoter HW, Ziem U, Spurny K, Huth F (1984). New results from implantation experiments with mineral fibres. Proceedings of a WHO/IARC Conference: Biological effects of man-made mineral fibres.

[9] Muller J, Delos M, Panin N, Rabolli V, Huaux F, Lison D (2009). Absence of carcinogenic response to multiwall carbon nanotubes in a 2-year bioassay in the peritoneal cavity of the rat. Toxicol Sci..

[10] Kim JS, Sung JH, Choi BG, Ryu HY, Song KS, Shin JH (2014). *In vivo* genotoxicity evaluation of lung cells from Fischer 344 rats following 28 days of inhalation exposure to MWCNTs, plus 28 days and 90 days post-exposure. Inhal Toxicol..

[11] Kato T, Totsuka Y, Ishino K, Matsumoto Y, Tada Y, Nakae D (2013). Genotoxicity of multi-walled carbon nanotubes in both *in vitro* and *in vivo* assay systems. Nanotoxicology..

[12] Jaurand MC, Bastie-Sigeac I, Renier A, Bignon J (1983). Comparative toxicities of different forms of asbestos on rat pleural mesothelial cells. Environ Health Perspect..

[13] Kenne K, Ljungquist S, Ringertz NR (1986). Effects of asbestos fibers on cell division, cell survival, and formation of thioguanine-resistant mutants in Chinese hamster ovary cells. Environ Res..

[14] Asakura M, Sasaki T, Sugiyama T, Takaya M, Koda S, Nagano K (2010). Genotoxicity and cytotoxicity of multi-wall carbon nanotubes in cultured Chinese hamster lung cells in comparison with chrysotile A fibers. J Occup Health..

[15] Muller J, Decordier I, Hoet PH, Lombaert N, Thomassen L, Huaux F (2008). Clastogenic and aneugenic effects of multi-wall carbon nanotubes in epithelial cells. Carcinogenesis..

[16] Oshimura M, Hesterberg TW, Tsutsui T, Barrett JC (1984). Correlation of asbestos-induced cytogenetic effects with cell transformation of Syrian hamster embryo cells in culture. Cancer Res..

[17] Craighead JE, Akley NJ, Gould LB, Libbus BL (1987). Characteristics of tumors and tumor cells cultured from experimental asbestos-induced mesotheliomas in rats. Am J Pathol..

[18] Jensen CG, Jensen LC, Rieder CL, Cole RW, Ault JG (1996). Long crocidolite asbestos fibers cause polyploidy by sterically blocking cytokinesis. Carcinogenesis..

[19] Sargent LM, Reynolds SH, Castranova V (2010). Potential pulmonary effects of engineered carbon nanotubes: *in vitro* genotoxic effects. Nanotoxicology..

[20] Sugimoto K, Senda-Murata K, Oka S (2008). Construction of three quadruple-fluorescent MDA435 cell lines that enable monitoring of the whole chromosome segregation process in the living state. Mutat Res..

[21] Phillips KK, Welch DR, Miele ME, Lee JH, Wei LL, Weissman BE (1996). Suppression of MDA-MB-435 breast carcinoma cell metastasis following the introduction of human chromosome 11. Cancer Res..

[22] Monteiro-Riviere NA, Nemanich RJ, Inman AO, Wang YY, Riviere JE (2005). Multi-walled carbon nanotube interactions with human epidermal keratinocytes. Toxicol Lett..

[23] Tabet L, Bussy C, Amara N, Setyan A, Grodet A, Rossi MJ (2009). Adverse effects of industrial multiwalled carbon nanotubes on human pulmonary cells. J Toxicol Environ Health A..

[24] Kagan VE, Tyurina YY, Tyurin VA, Konduru NV, Potapovich AI, Osipov AN (2006). Direct and indirect effects of single walled carbon nanotubes on RAW 264.7 macrophages: role of iron. Toxicol Lett..

[25] Yasui M, Koyama N, Koizumi T, Senda-Murata K, Takashima Y, Hayashi M (2010). Live cell imaging of micronucleus formation and development. Mutat Res..

